# Supplementary material for: Coagulation pretreatment could deteriorate reverse osmosis membrane fouling
Source: Nat Commun. 2026 Mar 19;17:4168. doi: 10.1038/s41467-026-70892-4 (PMC13153199; doi:10.1038/s41467-026-70892-4)
Supplement: Supplementary file 1 — Supplementary Information [file 41467_2026_70892_MOESM1_ESM.pdf]

Supplementary Information

Coagulation pretreatment could deteriorate reverse osmosis membrane fouling

Haojie Ding[1], Shuai Liang[2,*], Weichen Lin[1], Chao Chen[1], Ruonan Gao[2], Yufang Li[1], Ye Li[3], Kang Xiao[4,*], Xia Huang[1,*]

[1] State Key Laboratory of Regional Environment and Sustainability, School of Environment, Tsinghua University, Beijing 100084, China

[2] Beijing Key Lab for Source Control Technology of Water Pollution, College of Environmental Science and Engineering, Beijing Forestry University, Beijing 100083, China

[3] China Huaneng Group Clean Energy Research Institute Co., Ltd, Beijing 102209, China

[4] College of Resources and Environment, University of Chinese Academy of Sciences, Beijing 100049, China

---

[*]Corresponding authors

E-mail addresses: shuai_liang@bjfu.edu.cn (S. Liang), kxiao@ucas.ac.cn (K. Xiao), xhuang@tsinghua.edu.cn (X. Huang)

Totally 32 pages including 20 Figures, 8 Tables, and 3 Notes.

Contents

1. Supplementary Discussion

Supplementary Note 1. Theoretical estimation of concentration polarization (CP) effect. S4

Supplementary Note 2. The sequential order of functional group changes duringmembrane fouling based on the 2DCoS asynchronous maps. S6

Supplementary Note 3. Detailed procedures and discussions of the coagulation experiment....................S7

2. Supplementary Figures and Tables

Supplementary Figure 1. Schematic illustration of a typical RO-based zero liquid discharge (ZLD) process for industrial wastewater (e.g., desulfurization wastewater in coal-fired power plants) treatment. S9

Supplementary Figure 2. Variations of the filtration resistance of the different RO fouling layers in the Ctrl, Fe, and Al scenarios. S9

Supplementary Figure 3. The element ratios of different membrane surfaces determined by EDS spectra....................S10

Supplementary Table 1. Measured water quality indexes of the practical desulfurization wastewater....................S11

Supplementary Figure 4. The XPS on RO membrane samples of Ctrl (a), Al (b), and Fe (c) groups. S12

Supplementary Figure 5. FTIR spectra of the surface of fouled membrane samples in the Ctrl (a), Fe (b), and Al (c) scenarios. S13

Supplementary Figure 6. Asynchronous maps by two-dimensional correlation spectroscopy (2DCoS) for organic functional groups detected in the Ctrl (a), Al (b), and Fe (c) groups. S14

Supplementary Table 2. The 2DCoS data on the assignment and sign of cross peaks in synchronous (Φ) and asynchronous (Ψ, in parentheses) maps of membrane samples in the Ctrl group with increasing operating time. S14

Supplementary Table 3. The 2DCoS data on the assignment and sign of cross peaks in synchronous (Φ) and asynchronous (Ψ, in parentheses) maps of membrane samples in the Fe group with increasing operating time....................S15

Supplementary Table 4. The 2DCoS data on the assignment and sign of cross peaks in synchronous (Φ) and asynchronous (Ψ, in parentheses) maps of membrane samples in the Al group with increasing operating time....................S16

Supplementary Figure 7. Normalized 3D-EEM fluorescence spectra of the feed and extracted foulants from the RO membrane samples in the Ctrl, Fe, and Al scenarios....................S17

Supplementary Figure 8. Distribution of fluorescence quotient spectra of Al/Fe. S18

Supplementary Figure 9. CLSM images of the fouling layers. Color code:

magenta, nucleic acid; red, α-manno- or α-glucopyranosyl polysaccharides; green, protein; blue, β-D-glucopyranosyl polysaccharides. S19
Supplementary Figure 10. Contributions of fouling factors in two-pass RO systems at a coal-fired power plant. Bio, Ing, and Org refer to biological, inorganic, and organic fouling, respectively; I×O, B×O, and B×I indicate interactions between inorganic and organic, biological and organic, and biological and inorganic fouling factors, respectively; B×I×O represents the interaction of three types of fouling; Unexp is unexplained variance. S20
Supplementary Figure 11. The Chao1 (a) and Simpson (b) indexes of microbial community in the Feed, Ctrl, Fe, and Al scenarios. S20
Supplementary Figure 12. Relative abundances of dominant microbes at the family level. S21
Supplementary Figure 13. The relationships between microbial genera and environmental factors..S22
Supplementary Figure 14. Redundancy analysis of the factors (gray arrows) influencing the species (golden arrows). S23
Supplementary Table 5. Topological properties of microbial communities.....S24
Supplementary Table 6. Relative abundances of major gene prediciton pathways based on KEGG database at level 1. ....S25
Supplementary Figure 15. The relative abundance of functional genes related to metabolism. S26
Supplementary Figure 16. ROS levels (a), SOD activity (b), and CAT activity (c) of fouled RO membrane samples in the Ctrl, Al, and Fe groups on day 20. The error bars indicate standard deviation values from the triplicate samples. S27
Supplementary Table 7. Dosage of Fe and Al salt coagulants in the preliminary experiments. S28
Supplementary Figure 17. Effect of coagulant dosage on residual iron (a) and residual aluminum (b) in desulfurization wastewater; effect of dosage of iron (c) and aluminum (d) coagulants on TOC removal in desulfurization wastewater. .S29

3. Supplementary Methods
Supplementary Figure 18. Sampling schedule for the RO filtration experiment in each group. S30
Supplementary Table 8. Fluorescent stains and the observation parameters. ..S31
Supplementary Figure 19. Fluorescent dyeing protocol for CLSM detection. S31
Supplementary Figure 20. Flow chart of variance partitioning analysis in quantifying the individual and interactive contributions of various fouling types. S32

1. Supplementary Discussion

Supplementary Note 1. Theoretical estimation of concentration polarization (CP) effect.

To evaluate whether CP could significantly enrich residual coagulant ions at the membrane surface and thereby contribute to membrane fouling, the enrichment factor for each group was quantitatively estimated using classical boundary layer theory under identical hydrodynamic conditions. The enrichment factor is defined as:

$$F_{\mathrm{CP}} = \frac{C_{\mathrm{m}}}{C_{\mathrm{b}}} = \exp\left(\frac{J^*}{k}\right) \tag{S1}$$

where F_{CP} is the enrichment factor of CP, C_{m} denotes the solute concentration at the membrane surface (mg L^{-1}), C_{b} is the solute concentration in the bulk solution (mg L^{-1}), J^* refers to the membrane flux expressed in the International System of Units (m s^{-1}), and k is the mass transfer coefficient (m s^{-1}).

The mass transfer coefficient is given by:

$$k = \frac{D}{\delta} \tag{S2}$$

where D represents the molecular diffusion coefficient of the solute (m^2 s^{-1}), and δ is the thickness of the hydrodynamic boundary layer (m).

The boundary layer thickness δ is estimated as:

$$\delta = 0.1 \times \frac{d_{\mathrm{h}}}{\sqrt{Re}} \tag{S3}$$

The Reynolds number Re is defined as:

$$Re = \frac{\rho v d_{\mathrm{h}}}{\mu} \tag{S4}$$

The following parameters were used in the calculations: water density ρ = 1005 kg m^{-3}, dynamic viscosity μ = 8.9×10^{-4} Pa·s, crossflow velocity v = 0.06 m s^{-1},

hydraulic diameter $d_h = 1.35\times10^{-3}$ m, and membrane flux $J = 25$ L m^{-2} h^{-1} = 6.94×10^{-6} m s^{-1}.

The Reynolds number is calculated as:

$$Re = \frac{1005 \times 0.06 \times 1.35 \times 10^{-3}}{8.9 \times 10^{-4}} \approx 91.47 \tag{S5}$$

The boundary layer thickness is calculated as:

$$\delta = 0.1 \cdot \frac{1.35 \times 10^{-3}}{\sqrt{91.47}} \approx 1.40 \times 10^{-5}\ \mathrm{m} \tag{S6}$$

Using ion-specific diffusion coefficients for each group: $D_{Fe} = 7.0\times10^{-10}$ m^2 s^{-1} (Fe^{3+}), $D_{Al} = 7.0\times10^{-10}$ m^2 s^{-1} (Al^{3+}), and $D_{Ctrl} = 6.5\times10^{-10}$ m^2 s^{-1} (average of Fe^{3+} and Al^{3+}), the mass transfer coefficients are calculated as:

$$k_{\mathrm{Fe}} = \frac{7.0 \times 10^{-10}}{1.40 \times 10^{-5}} = 4.96 \times 10^{-5}\ \mathrm{m} \tag{S7}$$

$$k_{\mathrm{Al}} = \frac{6.0 \times 10^{-10}}{1.40 \times 10^{-5}} = 4.25 \times 10^{-5}\ \mathrm{m} \tag{S8}$$

$$k_{\mathrm{Ctrl}} = \frac{6.5 \times 10^{-10}}{1.40 \times 10^{-5}} = 4.64 \times 10^{-5}\ \mathrm{m} \tag{S9}$$

The CP enrichment factors are computed as:

$$\left.\frac{C_{\mathrm{m}}}{C_{\mathrm{b}}}\right|_{\mathrm{Fe}} = \exp\left(\frac{6.94 \times 10^{-6}}{4.96 \times 10^{-5}}\right) \approx \exp(0.140) \approx 1.15 \tag{S10}$$

$$\left.\frac{C_{\mathrm{m}}}{C_{\mathrm{b}}}\right|_{\mathrm{Al}} = \exp\left(\frac{6.94 \times 10^{-6}}{4.25 \times 10^{-5}}\right) \approx \exp(0.163) \approx 1.18 \tag{S11}$$

$$\left.\frac{C_{\mathrm{m}}}{C_{\mathrm{b}}}\right|_{\mathrm{Ctrl}} = \exp\left(\frac{6.94 \times 10^{-6}}{4.64 \times 10^{-5}}\right) \approx \exp(0.150) \approx 1.16 \tag{S12}$$

These similarly low CP enrichment factors suggest that CP is not a major contributor to the differences in membrane fouling observed among the three groups. The observed variations are more likely driven by other mechanisms, including interactions between residual coagulants and extracellular polymeric substances, shifts

in microbial communities, or other physicochemical processes at the membrane interface.

Supplementary Note 2. The sequential order of functional group changes duringmembrane fouling based on the 2DCoS asynchronous maps.

Ctrl group: 873 (C−N in Ar−NO_2) → 833 (C−H) → 1585 (Amide II) → 1240 (>P=O of biomass) → 1504 (C=C aromatic) → 1294 (C−O of carboxyl) → 690 (−OH of aromatic ring) → 1080 (C−O stretching of aromatic ether) → 1105 (C−O−C) → 1169 (C−C) → 1013 cm^{-1} (C−C).

Al group: 1487 (Amines) > 1504 (C=C aromatic) > 1077 (C−O stretching of aromatic ether) > 1240 (>P=O of biomass) > 1740, 1860 (vibration of C=O in −COOH in sialic acids, C=O) > 1585 (Amide II) > 750 (−OH of aromatic ring) > 872 (C−N in Ar−NO_2) > 1013 (C−C) > 1638 cm^{-1} (Amide I).

Fe group: 1240 (>P=O of biomass) → 1504 (>C=C aromatic) → 1585 (Amide II) → 1487 (Amines) → 873 (C−N in Ar−NO_2) → 942 (R−NO_2) → 833 (C−H) → 1013 (C−C) → 1740, 1860 (vibration of C=O in −COOH in sialic acids, C=O) → 690, 1079 (−OH of aromatic ring, =C−O stretching of aromatic ether) → 1658 (Amide I) cm^{-1}.

Supplementary Note 3. Detailed procedures and discussions of the coagulation experiment.

Ferric chloride ($FeCl_3$), polymeric ferric sulfate (PFS), aluminum chloride ($AlCl_3$), and aluminum chlorohydrate (PAC) were selected as typical iron and aluminum salt coagulants for the preliminary experiments. All chemical agents were of analytical grade and were purchased from Macklin Inc. (Shanghai, China). The specific dosages of each coagulant are listed in Table S7. Note that in the Fe salt group, $FeCl_3$ and PFS were adjusted to the same iron content, and similarly, the Al salt group had equivalent aluminum content.

After the coagulants were added to the desulfurization wastewater, the coagulation experiments were performed at a rapid stirring speed of 100 rpm for 1 min to ensure uniform mixing. Then, stirring was reduced to 40 rpm for 15 min to promote floc growth. After a sedimentation period of 30 min, samples were taken 2 cm below the liquid surface to determine the residual iron or aluminum content and TOC. All experiments were conducted at least in duplicate at room temperature (25 ± 2 °C).

As shown in Figure S17a,b, there is an increasing trend in residual metals with increasing dosage of coagulants in both the Fe and Al salt groups. When the dosage range is below 8.61 mg L^{-1}, the residual iron concentrations of $FeCl_3$ and PFS are approximately coincide. When the dosage gradually increases, the residual iron of $FeCl_3$ increases more than that of PFS. The Al salt group also has a critical concentration at a dosage of 6.06 mg L^{-1}.

The TOC removal rates of $FeCl_3$ and PFS were 15.88% and 16.01%, respectively, when the Fe salt dosage was 8.61 mg L^{-1} (Figure S17c,d). However, further increasing the dose to 20.66 mg L^{-1} increased the TOC removal rate by only 0.62% and 4.05%, respectively. The Al and Fe salt groups showed similar trends in TOC removal.

The above results suggest that the high-dose Fe and Al salt groups cannot effectively promote TOC removal, and there is also a problem of high concentration of metal residues. In view of this, 5.16 mg L^{-1} $FeCl_3$ (calculated as iron content) and 5.05 mg L^{-1} $AlCl_3$ (calculated as aluminum content) were selected as coagulants for desulfurization wastewater in the low concentration range. At this dose, the residual iron concentration in the Fe salt group was 2.59 mg L^{-1} and the TOC removal rate was 12.03%; the residual aluminum concentration in the Al salt group was 2.36 mg L^{-1} and the TOC removal rate was 11.00%. All water samples were adjusted to near neutral pH with NaOH solution before RO filtration tests, and detailed water quality parameters can be found in Table S1.

2. Supplementary Figures and Tables

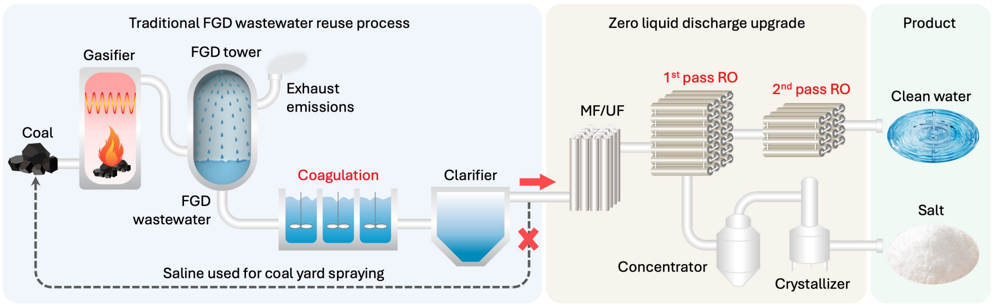

Supplementary Figure 1. Schematic illustration of a typical RO-based zero liquid discharge (ZLD) process for industrial wastewater (e.g., desulfurization wastewater in coal-fired power plants) treatment.

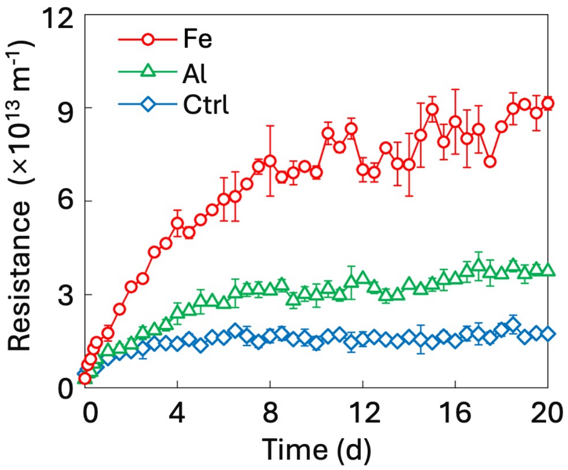

Supplementary Figure 2. Variations of the filtration resistance of the different RO fouling layers in the Ctrl, Fe, and Al scenarios. Error bars in the figure represent the s.d. (n = 3) and data are presented as mean values ± s.d.

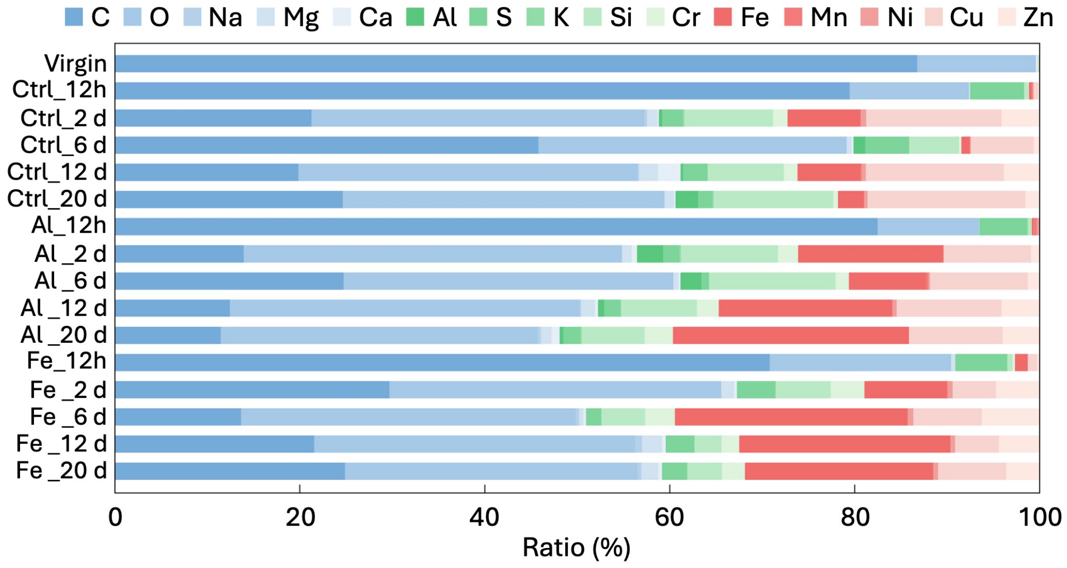

Supplementary Figure 3. The element ratios of different membrane surfaces determined by EDS spectra.

Supplementary Table 1. Measured water quality indexes of the practical desulfurization wastewater.

Index	Raw	Ctrl	Al	Fe
Turbidity (NTU)	282.5 ± 1.5	0.88 ± 0.08	0.91 ± 0.01	0.90 ± 0.04
Conductivity (mS cm^{-1})	23.09 ± 1.41	23.12 ± 0.62	23.25 ± 0.57	23.57 ± 0.06
pH	6.51 ± 0.01	7.10 ± 0.11	6.84 ± 0.06	6.98 ± 0.07
DOC (mg L^{-1})	14.55 ± 0.71	12.99 ± 0.29	12.63 ± 0.53	12.52 ± 0.34
Na^{+} (mg L^{-1})	864.53 ± 21.37	887.99 ± 13.13	882.60 ± 28.20	874.38 ± 46.03
K^{+} (mg L^{-1})	210.26 ± 9.55	224.86 ± 2.63	295.48 ± 10.62	291.79 ± 6.39
Ca^{2+} (mg L^{-1})	747.50 ± 15.61	678.60 ± 9.24	658.51 ± 7.20	650.45 ± 4.36
Mg^{2+} (g L^{-1})	1.93 ± 0.25	1.64 ± 0.15	1.64 ± 0.12	1.63 ± 0.09
F^{-} (mg L^{-1})	63.55 ± 5.74	66.98 ± 2.18	74.23 ± 5.90	69.14 ± 3.49
Cl^{-} (g L^{-1})	13.00 ± 1.68	13.37 ± 0.49	13.48 ± 0.35	13.34 ± 0.48
SO_4^{2-} (g L^{-1})	16.20 ± 2.20	16.09 ± 0.20	15.94 ± 0.05	15.91 ± 0.22
Al (mg L^{-1})	0.95 ± 0.09	0.63 ± 0.13	2.36 ± 0.30	0.49 ± 0.04
Fe (mg L^{-1})	0.51 ± 0.20	0.15 ± 0.01	0.26 ± 0.07	2.59 ± 0.10
Cu (mg L^{-1})	1.25 ± 0.57	0.78 ± 0.12	0.49 ± 0.05	1.03 ± 0.25
Si (mg L^{-1})	45.93 ± 1.32	29.93 ± 0.97	39.71 ± 1.46	21.55 ± 0.87
Mn (mg L^{-1})	22.66 ± 0.26	11.85 ± 0.49	15.53 ± 0.61	13.81 ± 0.55
Cr (mg L^{-1})	0.09 ± 0.04	0.07 ± 0.01	0.04 ± 0.01	0.04 ± 0.01

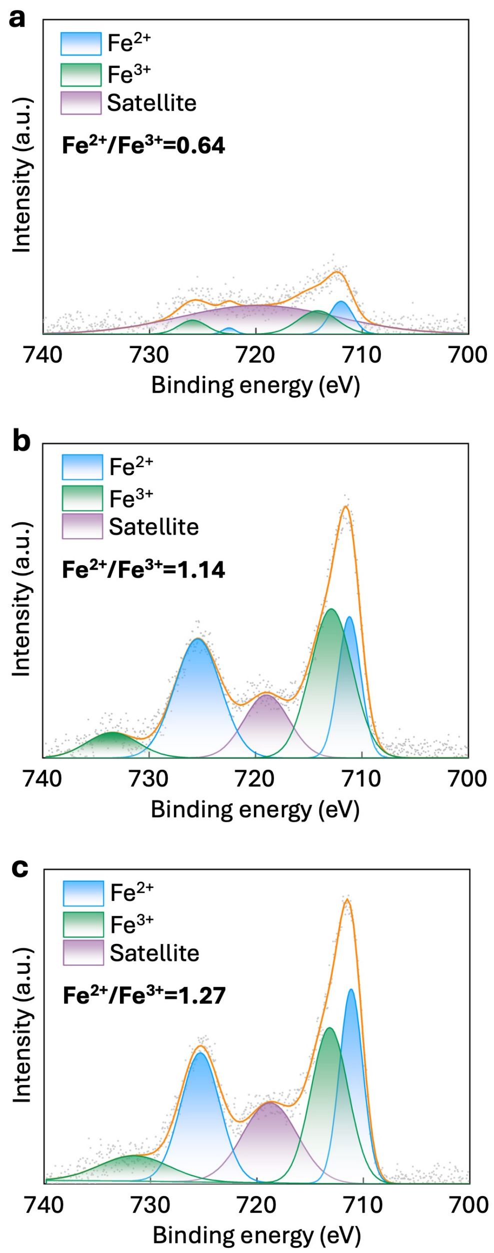

Supplementary Figure 4. The XPS on RO membrane samples of Ctrl (**a**), Al (**b**), and Fe (**c**) groups.

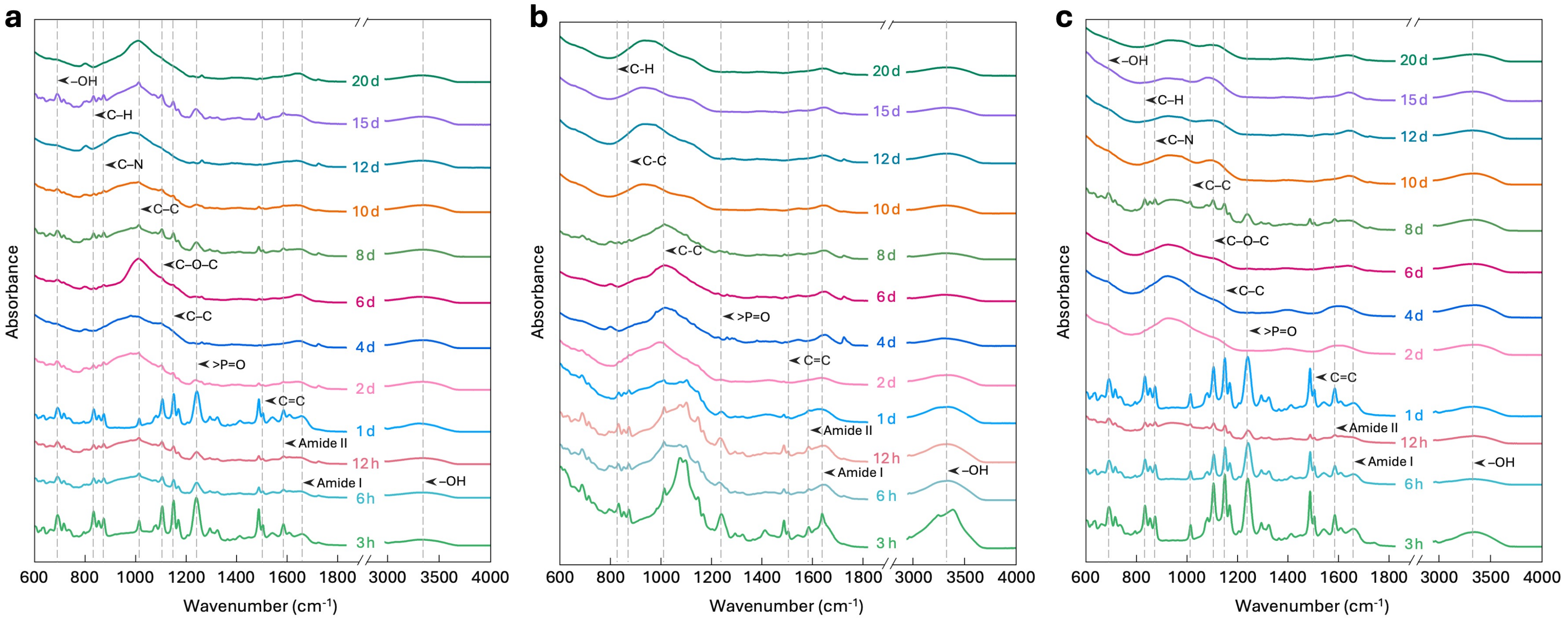

Supplementary Figure 5. FTIR spectra of the surface of fouled membrane samples in the Ctrl (**a**), Fe (**b**), and Al (**c**) scenarios.

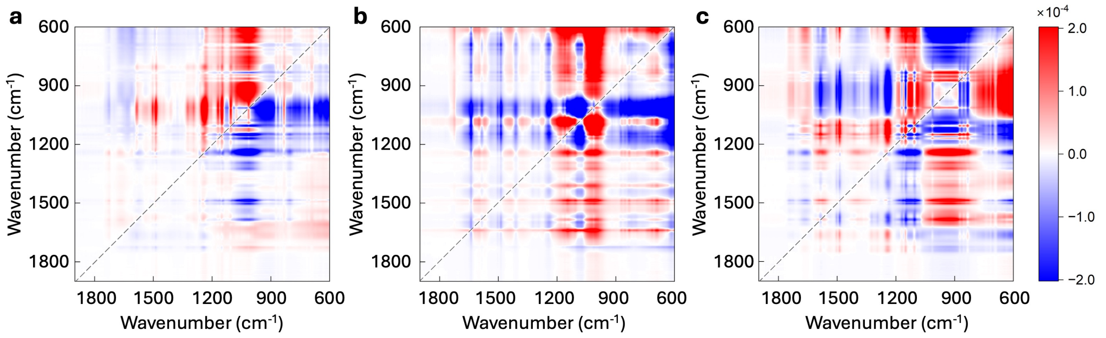

Supplementary Figure 6. Asynchronous maps by two-dimensional correlation spectroscopy (2DCoS) for organic functional groups detected in the Ctrl (**a**), Al (**b**), and Fe (**c**) groups.

Supplementary Table 2. The 2DCoS data on the assignment and sign of cross peaks in synchronous (Φ) and asynchronous (Ψ, in parentheses) maps of membrane samples in the Ctrl group with increasing operating time.

Peak (cm^{-1})	Assignment	Sign [a]										
		690	833	873	1013	1080	1105	1169	1240	1294	1504	1585
690	−OH of aromatic ring	+	+(−)	+(−)	+(+)	+(+)	+(+)	+(+)	+(−)	+(−)	+(−)	+(−)
833	C−H		+	+(−)	+(+)	+(+)	+(+)	+(+)	+(+)	+(+)	+(+)	+(+)
873	C−N in Ar−NO_2			+	+(+)	−(+)	−(+)	−(+)	−(+)	−(+)	−(+)	−(+)
1013	C−C				+	−(+)	−(+)	−(+)	−(+)	−(+)	−(+)	−(+)
1080	C−O stretching of aromatic ether					+	+(+)	+(+)	+(+)	+(+)	+(+)	+(+)
1105	C−O−C						+	+(+)	+(+)	+(+)	+(+)	+(−)
1169	C−C							+	+(+)	+(+)	+(+)	+(+)
1240	>P=O of biomass								+	+(+)	+(+)	+(−)
1294	C−O of carboxyl									+	+(−)	+(−)
1504	C=C aromatic										+	+(−)
1585	Amide II											+

[a] "+" means a positive sign and "−" means a negative sign.

Supplementary Table 3. The 2DCoS data on the assignment and sign of cross peaks in synchronous (Φ) and asynchronous (Ψ, in parentheses) maps of membrane samples in the Fe group with increasing operating time.

Peak (cm^{-1})	Assignment	Sign [a]												
		690	833	873	942	1013	1079	1240	1487	1504	1585	1658	1740	1860
690	−OH of aromatic ring	+	+(−)	+(−)	+(−)	+(−)	+(0)	+(−)	+(−)	+(−)	+(−)	+(+)	+(−)	+(−)
833	C−H		+	+(−)	+(−)	+(+)	+(+)	+(−)	+(−)	+(−)	+(−)	+(+)	+(+)	+(+)
873	C−N in Ar−NO_2			+	+(+)	+(+)	+(+)	+(−)	+(−)	+(−)	+(−)	+(+)	+(+)	+(+)
942	R−NO_2				+	+(+)	+(+)	+(−)	+(−)	+(−)	+(−)	+(+)	+(+)	+(+)
1013	C−C					+	+(+)	+(−)	+(−)	+(−)	+(−)	+(+)	+(+)	+(+)
1079	C−O stretching of aromatic ether						+	+(−)	+(−)	+(−)	+(−)	+(+)	+(+)	+(+)
1240	>P=O of biomass							+	+(+)	+(+)	+(+)	+(−)	+(−)	+(−)
1487	Amines								+	+(−)	+(−)	+(−)	+(0)	+(0)
1504	C=C aromatic									+	+(+)	+(−)	+(0)	+(0)
1585	Amide II										+	+(−)	+(+)	+(+)
1658	Amide I, C=O stretching vibrations											+	+(0)	+(0)
1740	Vibration of C=O in −COOH in sialic acids												+	+(0)
1860	C=O													+

[a] "+" means a positive sign, "–" means a negative sign, and "0" means a zero correlation.

Supplementary Table 4. The 2DCoS data on the assignment and sign of cross peaks in synchronous (Φ) and asynchronous (Ψ, in parentheses) maps of membrane samples in the Al group with increasing operating time.

Peak (cm^{-1})	Assignment	Sign [a]										
		750	872	1013	1077	1240	1487	1504	1585	1638	1740	1860
750	−OH of aromatic ring	+	+(+)	+(+)	+(−)	+(−)	+(−)	+(−)	+(−)	+(+)	+(−)	+(−)
872	C−N in Ar−NO_2		+	+(+)	+(−)	+(−)	+(−)	+(−)	+(−)	+(+)	+(−)	+(−)
1013	C−C			+	+(−)	+(−)	+(−)	+(−)	+(−)	+(+)	+(−)	+(−)
1077	C−O stretching of aromatic ether				+	+(+)	+(−)	+(−)	+(+)	+(+)	+(+)	+(+)
1240	>P=O of biomass					+	+(−)	+(−)	+(+)	+(+)	+(+)	+(+)
1487	Amines						+	+(+)	+(+)	+(+)	+(0)	+(0)
1504	C=C aromatic							+	+(+)	+(+)	+(+)	+(0)
1585	Amide II								+	+(−)	+(−)	+(−)
1638	Amide I, C=O stretching vibrations									+	+(0)	+(0)
1740	Vibration of C=O in −COOH in sialic acids										+	+(0)
1860	C=O											+

[a] "+" means a positive sign, "–" means a negative sign, and "0" means a zero correlation.

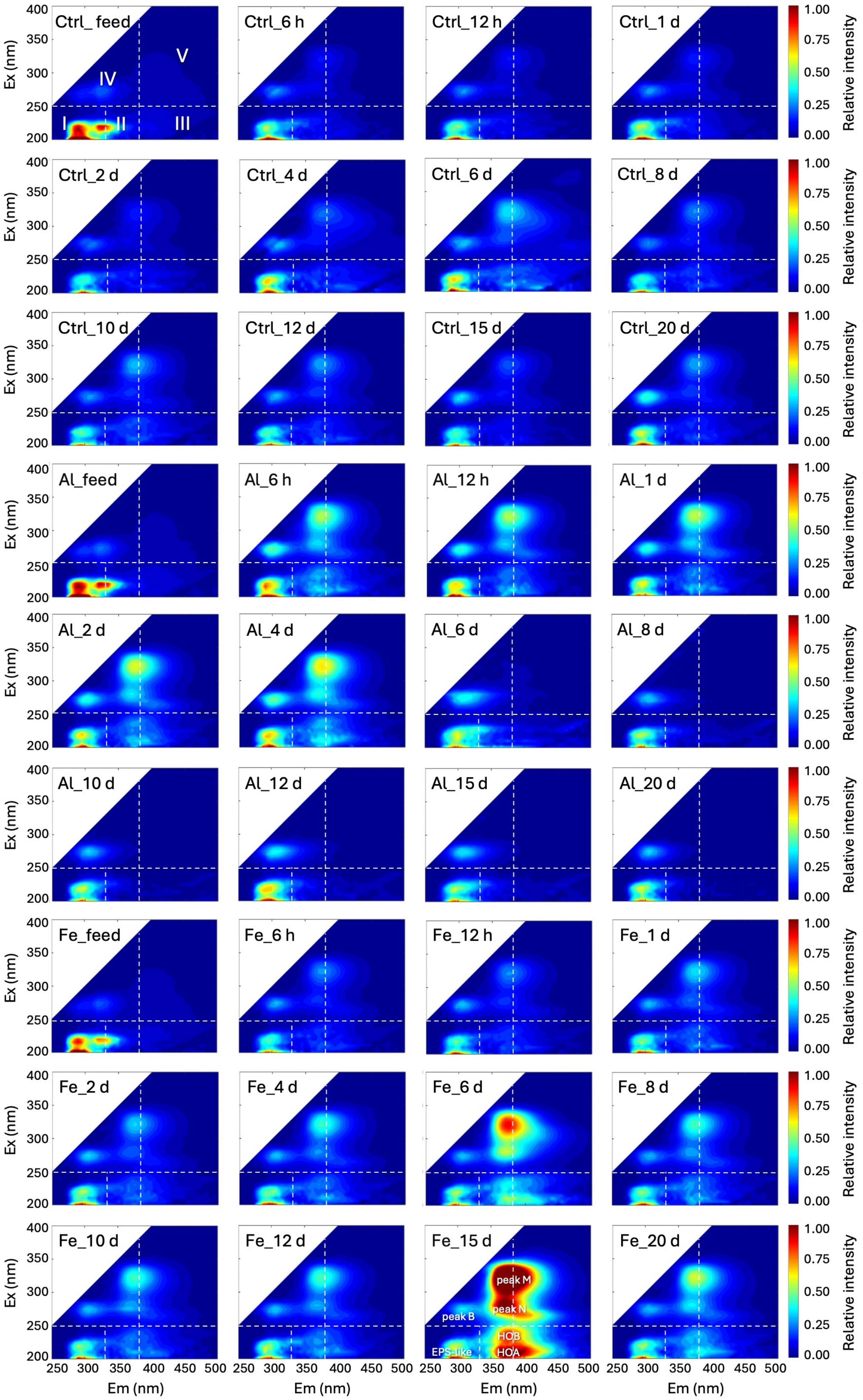

Supplementary Figure 7. Normalized 3D-EEM fluorescence spectra of the feed and extracted foulants from the RO membrane samples in the Ctrl, Fe, and Al scenarios.

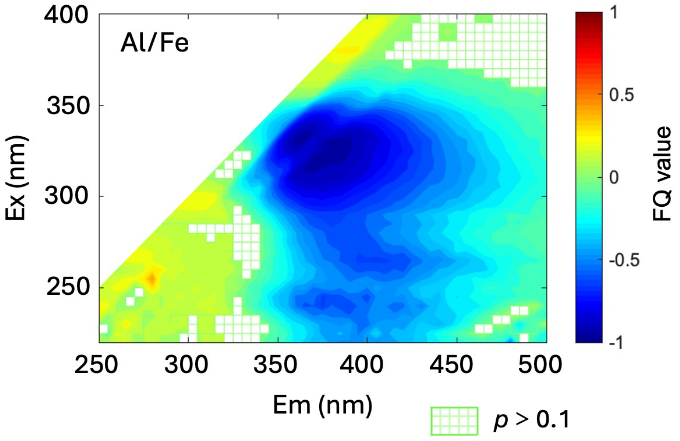

Supplementary Figure 8. Distribution of fluorescence quotient spectra of Al/Fe.

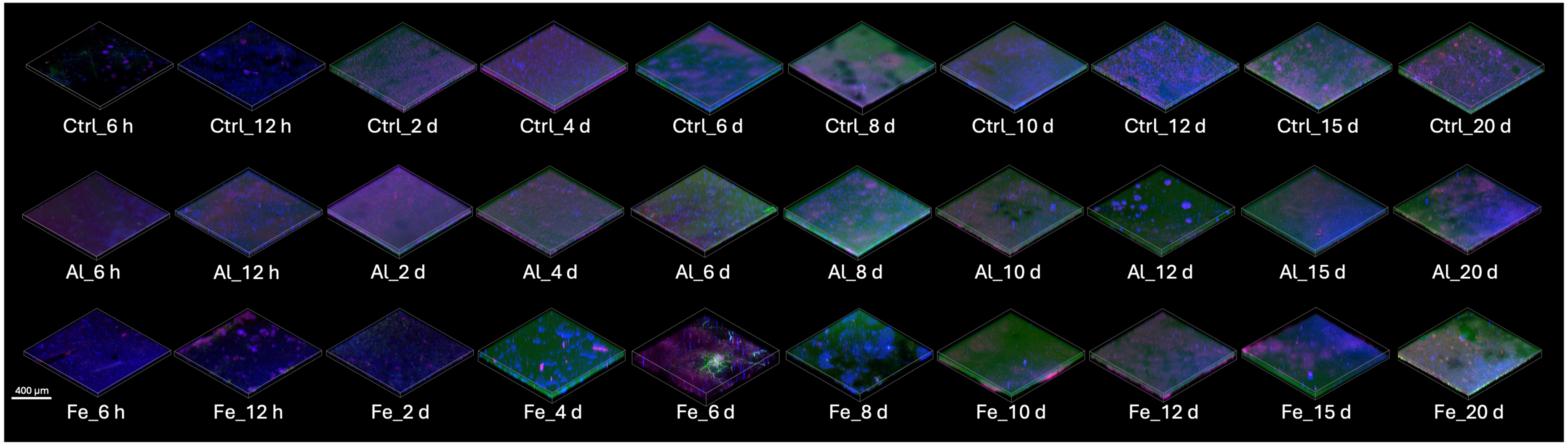

Supplementary Figure 9. CLSM images of the fouling layers. Color code: magenta, nucleic acid; red, *α*-manno- or *α*-glucopyranosyl polysaccharides; green, protein; blue, *β*-D-glucopyranosyl polysaccharides.

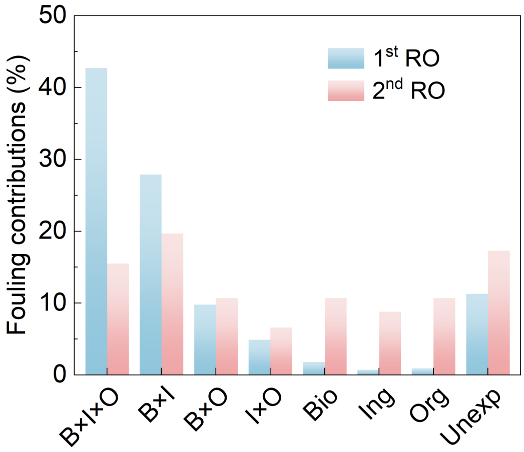

Supplementary Figure 10. Contributions of fouling factors in two-pass RO systems at a coal-fired power plant. Bio, Ing, and Org refer to biological, inorganic, and organic fouling, respectively; I×O, B×O, and B×I indicate interactions between inorganic and organic, biological and organic, and biological and inorganic fouling factors, respectively; B×I×O represents the interaction of three types of fouling; Unexp is unexplained variance.

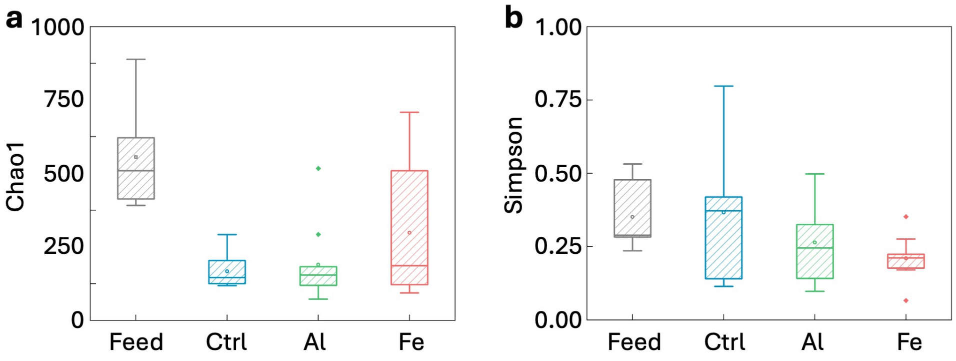

Supplementary Figure 11. The Chao1 (**a**) and Simpson (**b**) indexes of microbial community in the Feed, Ctrl, Fe, and Al scenarios. Box plots show the median, the 25th and 75th percentiles, and whiskers extending to 1.5× the interquartile range; individual points represent independent samples (n = 10 per group).

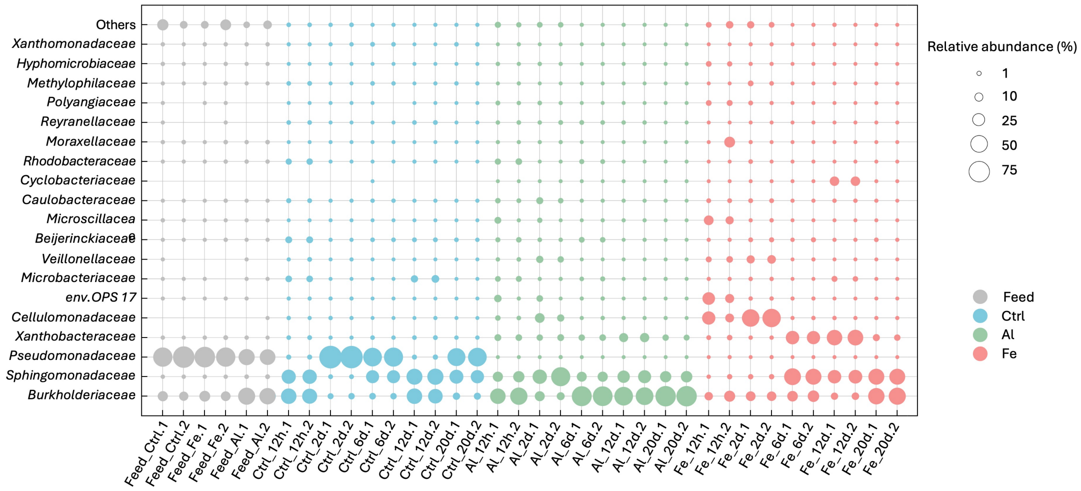

Supplementary Figure 12. Relative abundances of dominant microbes at the family level.

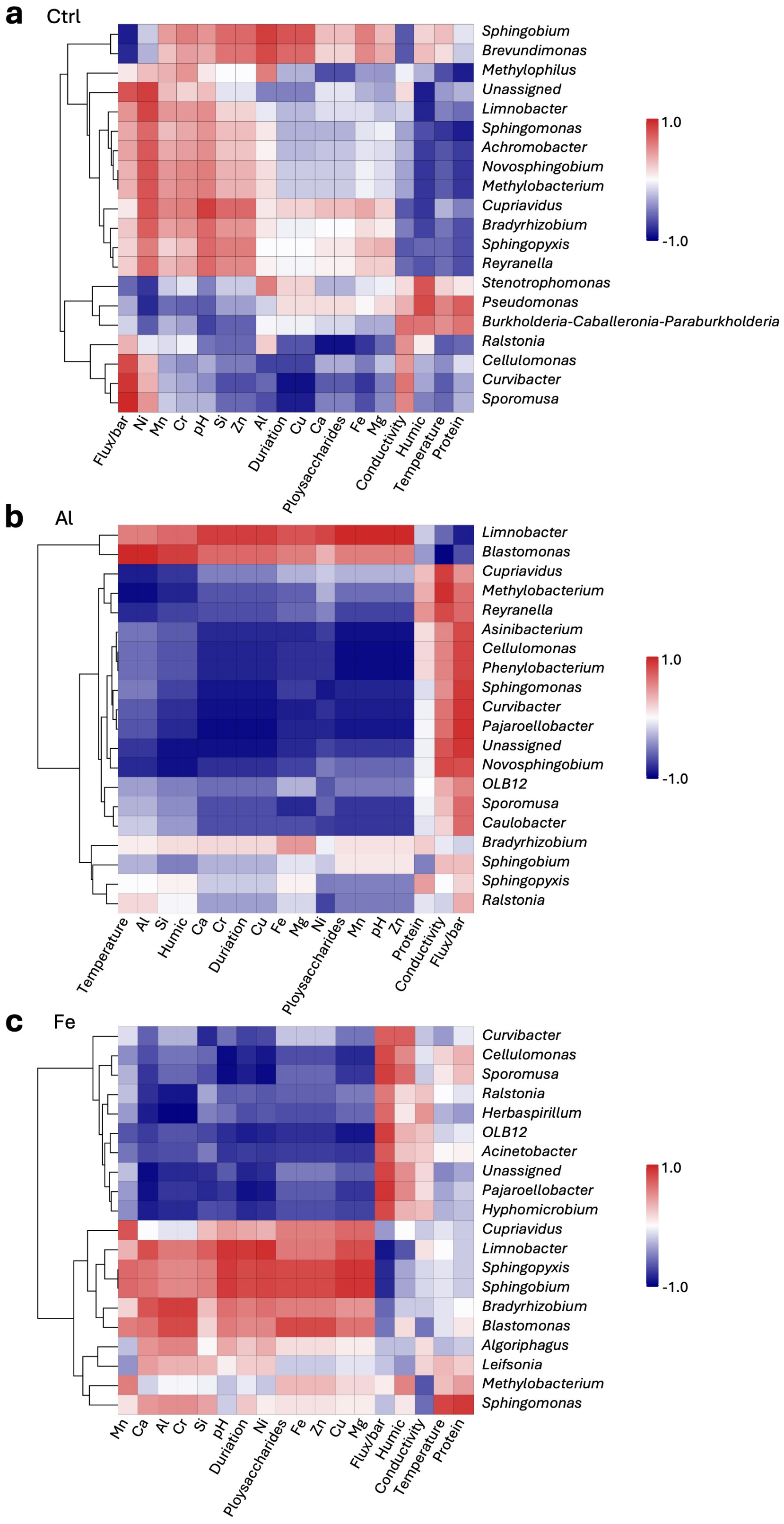

Supplementary Figure 13. The relationships between microbial genera and environmental factors.

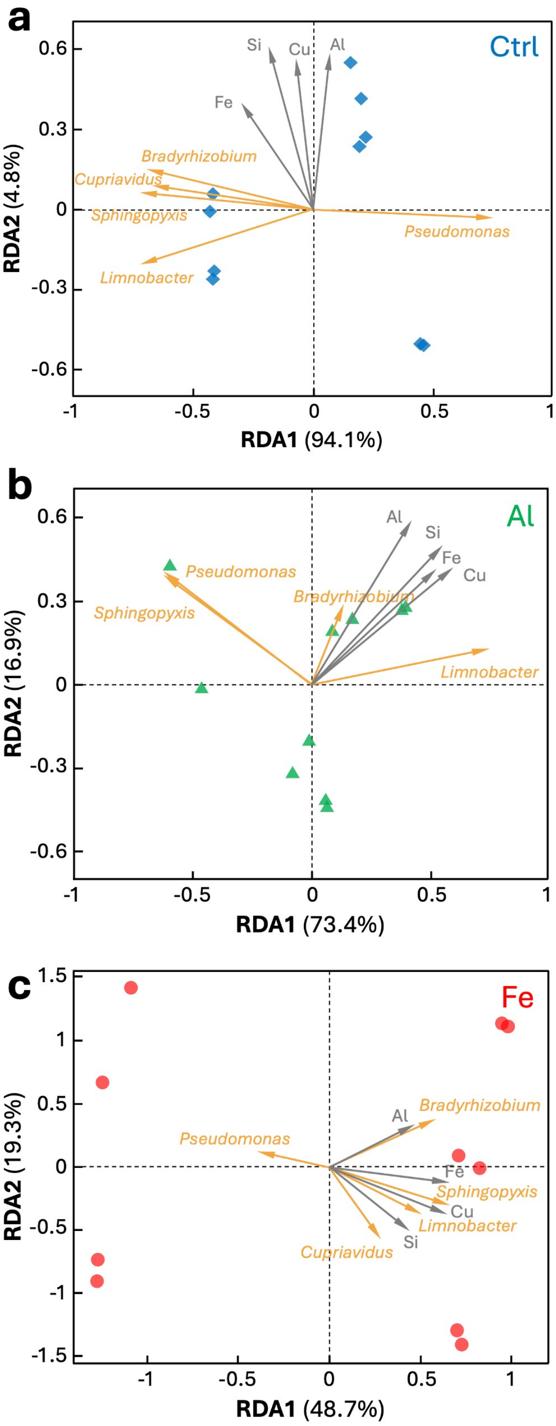

Supplementary Figure 14. Redundancy analysis of the factors (gray arrows) influencing the species (golden arrows).

Supplementary Table 5. Topological properties of microbial communities.

Network indexes	Ctrl	Al	Fe
Similarity threshold (St)	0.891	0.895	0.908
Total nodes (n)	139	113	125
Total links	253	142	178
R^2 of power-law	0.865	0.816	0.814
Efficiency	0.968	0.944	0.974
Average degree (avgK)	3.64	2.513	2.848
Average clustering coefficient (avgCC)	0.203	0.188	0.173
Average path distance (GD)	5.609	4.341	5.215
Geodesic efficiency (E)	0.252	0.323	0.250
Modularity	0.629	0.728	0.734
Module	20	17	21
Density	0.026	0.022	0.023
Reciprocity	1	1	1
Transitivity	0.343	0.253	0.277
Connectedness	0.616	0.272	0.604
Maximal degree	15	8	8
Nodes with max degree	OTU_459	OTU_1; OTU_6	OTU_155; OTU_61
Centralization of degree (CD)	0.084	0.050	0.042
Maximal betweenness	2616.13	435.933	1250.45
Nodes with max betweenness	OTU_304	OTU_83	OTU_29
Centralization of betweenness (CB)	0.258	0.063	0.144
Maximal stress centrality	7334	1064	3203
Nodes with max stress centrality	OTU_304	OTU_1	OTU_29
Centralization of stress centrality (CS)	0.723	0.154	0.364
Maximal eigenvector centrality	0.301	0.399	0.381
Nodes with maximal eigenvector centrality	OTU_41	OTU_6	OTU_58
Centralization of eigenvector centrality (CE)	0.265	0.358	0.348

Supplementary Table 6. Relative abundances of major gene prediciton pathways based on KEGG database at level 1.

Group	Metabolism	Environmental Information Processing	Cellular Processes	Genetic Information Processing	Human Diseases	Organismal Systems
Ctrl	47.32 ± 0.011	18.07 ± 0.043	13.81 ± 0.017	10.65 ± 0.005	6.16 ± 0.001	3.99 ± 0.033
Al	47.25 ± 0.004	16.52 ± 0.014	14.06 ± 0.099	11.50 ± 0.118	6.00 ± 0.005	4.67 ± 0.015
Fe	48.53 ± 0.143	15.05 ± 0.218	13.82 ± 0.212	10.71 ± 0.156	6.67 ± 0.076	5.22 ± 0.054

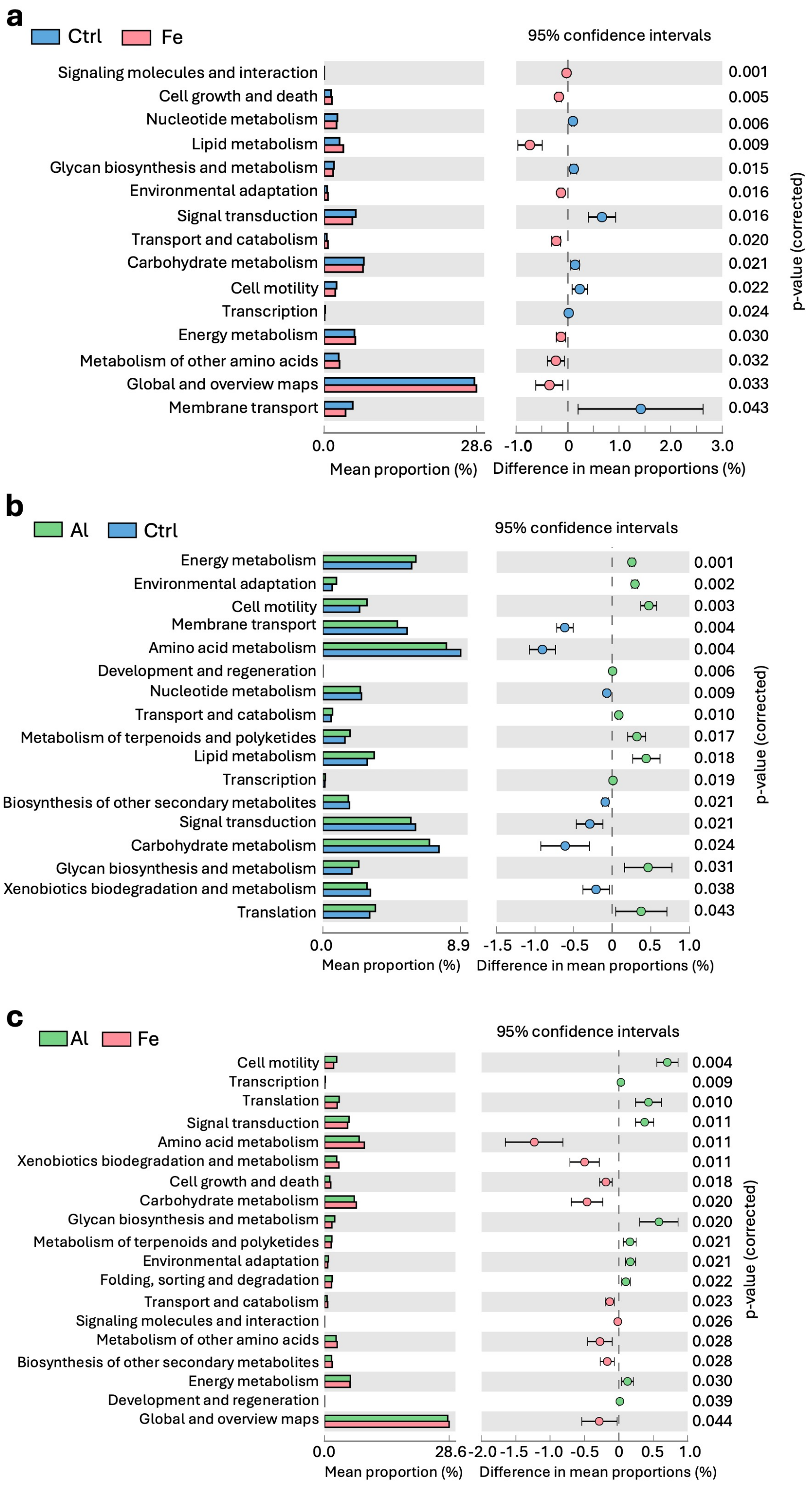

Supplementary Figure 15. The relative abundance of functional genes related to metabolism.

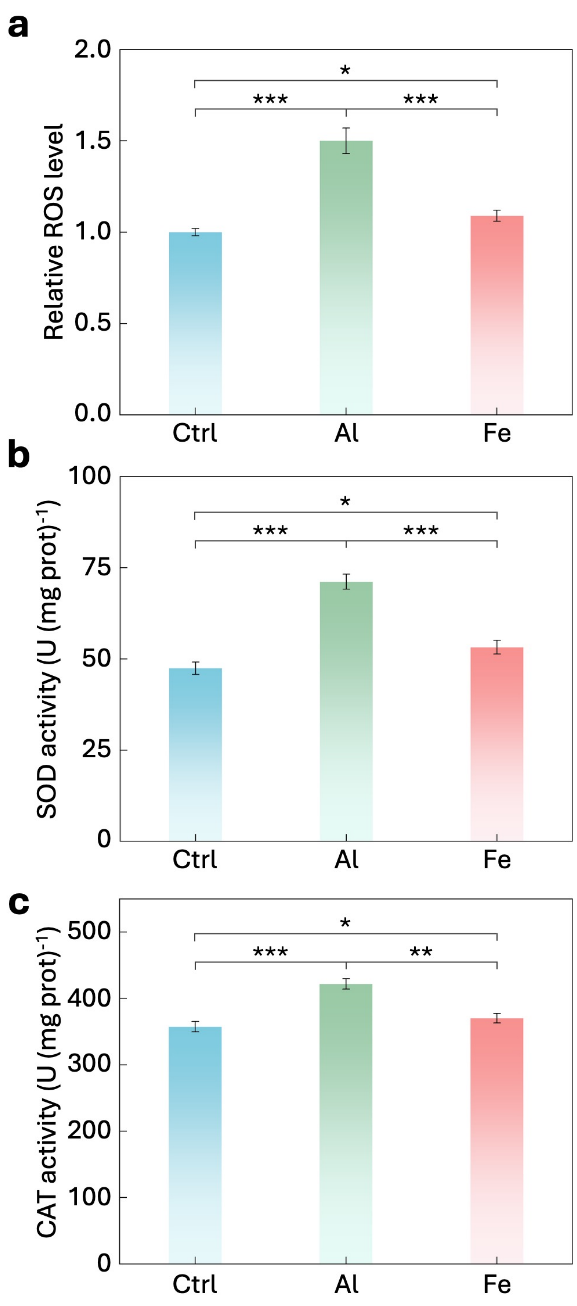

Supplementary Figure 16. ROS levels (**a**), SOD activity (**b**), and CAT activity (**c**) of fouled RO membrane samples in the Ctrl, Al, and Fe groups on day 20. The error bars indicate standard deviation values from the triplicate samples. Error bars in the figure represent the s.d. (n = 3) and data are presented as mean values ± s.d.

Supplementary Table 7. Dosage of Fe and Al salt coagulants in the preliminary experiments.

$FeCl_3$ dosage (mg L^{-1})	PFS dosage (mg L^{-1})	Calculated as Fe content (mg L^{-1})	$AlCl_3$ dosage (mg L^{-1})	PAC dosage (mg L^{-1})	Calculated as Al content (mg L^{-1})
5	8.2	1.72	5	14.6	1.01
10	16.4	3.44	10	29.4	2.02
15	24.6	5.16	15	44.0	3.03
20	32.8	6.89	20	58.8	4.04
25	41.0	8.61	25	73.4	5.05
30	49.2	10.33	30	88.4	6.06
40	65.6	13.77	40	117.6	8.08
50	82.0	17.22	50	147.0	10.11
60	98.4	20.66	60	176.2	12.13

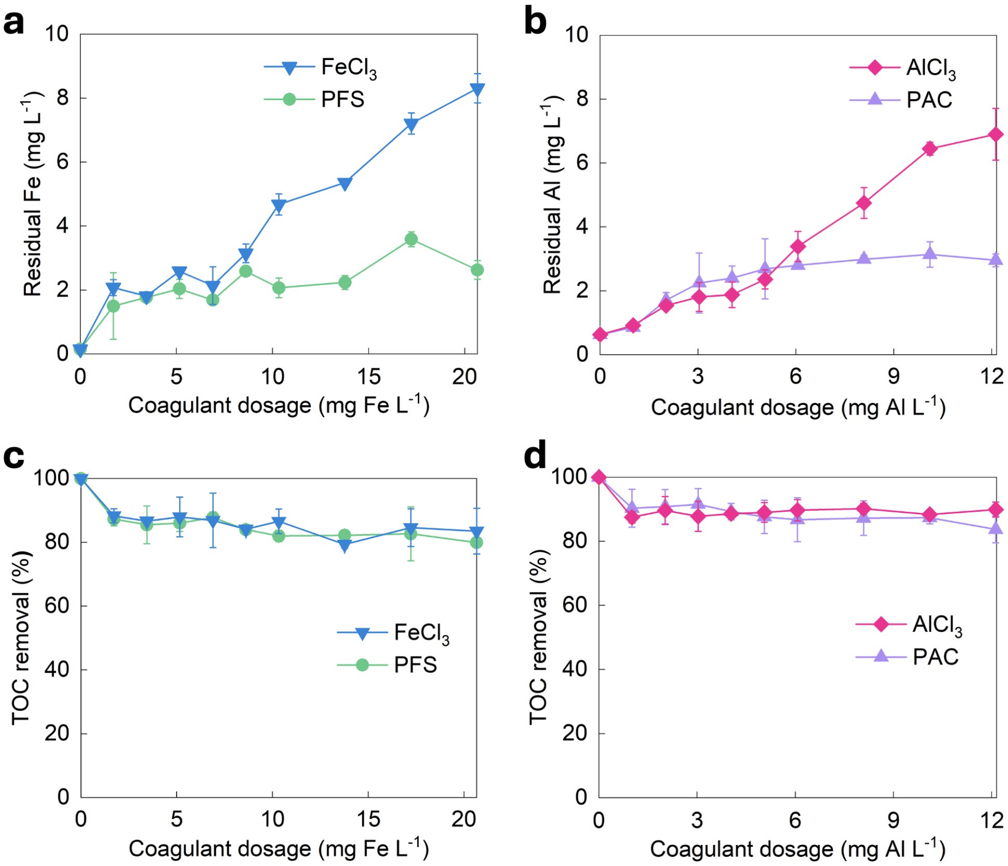

Supplementary Figure 17. Effect of coagulant dosage on residual iron (**a**) and residual aluminum (**b**) in desulfurization wastewater; effect of dosage of iron (**c**) and aluminum (**d**) coagulants on TOC removal in desulfurization wastewater. Error bars in the figure represent the s.d. (n = 3) and data are presented as mean values ± s.d.

3. Supplementary Methods

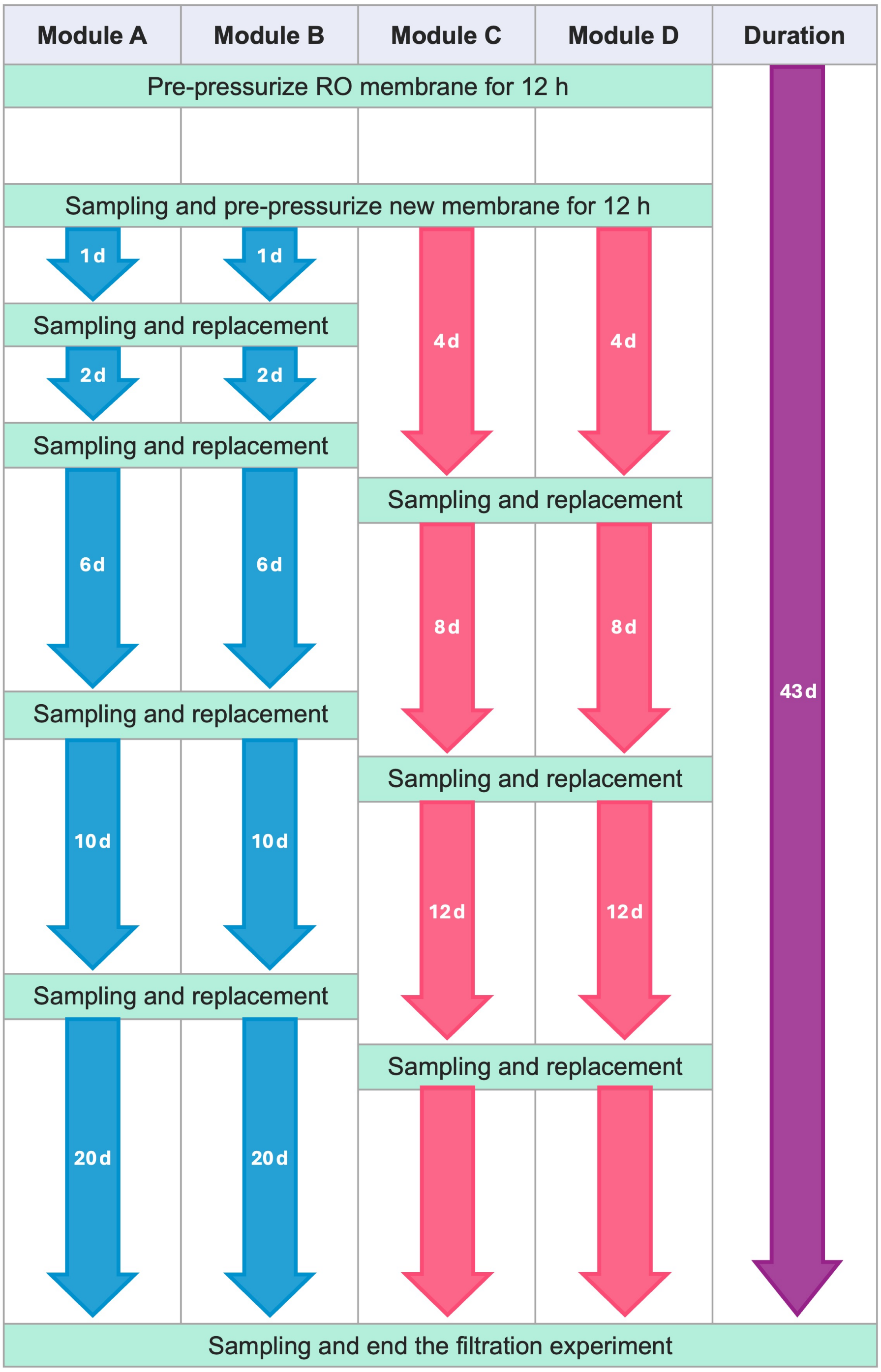

Supplementary Figure 18. Sampling schedule for the RO filtration experiment in each group.

Supplementary Table 8. Fluorescent stains and the observation parameters.

Stain	Excitation (nm)	Emission (nm)	Specificity	Color
PI	633	641–718	Dead cells	Magenta
Concanavalin A	561	565–609	α-mannopyranosyl and α-glucopyranosyl sugar	Red
FITC	488	500–541	Amine-reactive matters, such as protein and amino sugars	Green
Calcofluor White	405	410–483	β-D-glucopyranosyl sugar, such as cellulose in cell wall	Blue

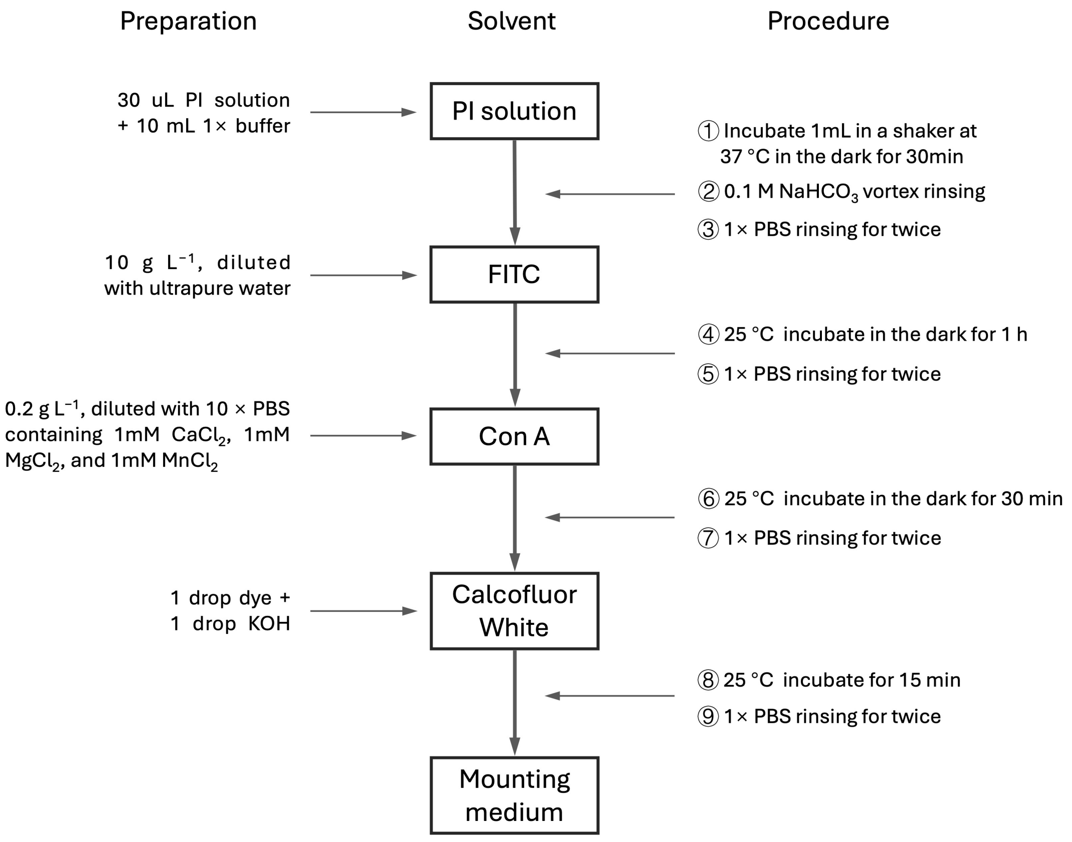

Supplementary Figure 19. Fluorescent dyeing protocol for CLSM detection.

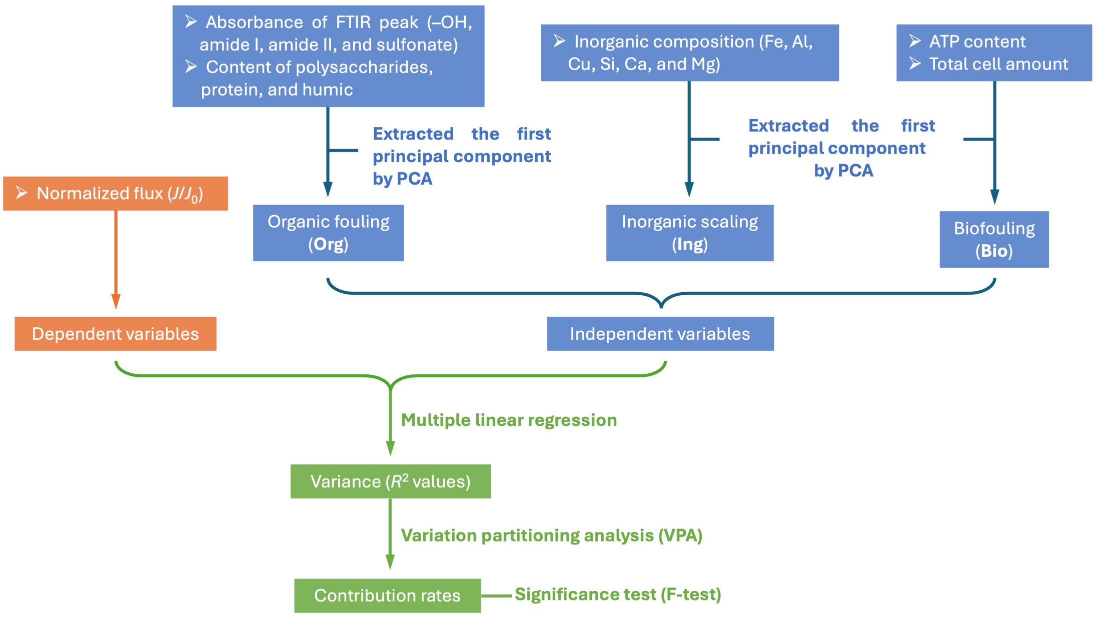

Supplementary Figure 20. Flow chart of variance partitioning analysis in quantifying the individual and interactive contributions of various fouling types.
