# Supplementary material for: Coagulation pretreatment could deteriorate reverse osmosis membrane fouling
Source: Nat Commun. 2026 Mar 19;17:4168. doi: 10.1038/s41467-026-70892-4 (PMC13153199; doi:10.1038/s41467-026-70892-4)
Supplement: Supplementary file 2 — Reporting summary [file 41467_2026_70892_MOESM2_ESM.pdf]

nature portfolio

Corresponding author(s): Xia Huang
Last updated by author(s): Feb 6, 2026

Reporting Summary

Nature Portfolio wishes to improve the reproducibility of the work that we publish. This form provides structure for consistency and transparency in reporting. For further information on Nature Portfolio policies, see our Editorial Policies and the Editorial Policy Checklist.

Statistics

For all statistical analyses, confirm that the following items are present in the figure legend, table legend, main text, or Methods section.

n/a	Confirmed	
☐	☒	The exact sample size (*n*) for each experimental group/condition, given as a discrete number and unit of measurement
☐	☒	A statement on whether measurements were taken from distinct samples or whether the same sample was measured repeatedly
☐	☒	The statistical test(s) used AND whether they are one- or two-sided<br>*Only common tests should be described solely by name; describe more complex techniques in the Methods section.*
☐	☒	A description of all covariates tested
☐	☒	A description of any assumptions or corrections, such as tests of normality and adjustment for multiple comparisons
☐	☒	A full description of the statistical parameters including central tendency (e.g. means) or other basic estimates (e.g. regression coefficient) AND variation (e.g. standard deviation) or associated estimates of uncertainty (e.g. confidence intervals)
☐	☒	For null hypothesis testing, the test statistic (e.g. *F*, *t*, *r*) with confidence intervals, effect sizes, degrees of freedom and *P* value noted<br>*Give P values as exact values whenever suitable.*
☒	☐	For Bayesian analysis, information on the choice of priors and Markov chain Monte Carlo settings
☒	☐	For hierarchical and complex designs, identification of the appropriate level for tests and full reporting of outcomes
☐	☒	Estimates of effect sizes (e.g. Cohen's *d*, Pearson's *r*), indicating how they were calculated

Our web collection on statistics for biologists contains articles on many of the points above.

Software and code

Policy information about availability of computer code

Data collection	No software was used for data collection.
Data analysis	2DCoS spectra: 2DShige (Version 1.3)<br>3D-EEM: Matlab (Version R2019a)<br>FQ and prominent region distributions: Matlab (Version R2019a)<br>PCR: Gene tools analysis (Version5.0, SynGene)<br>DNA sequencing analysis: BLASTP (Version 2.2.31+); BBMap (Version 39.01)<br>Visualization of ecological interactive networks: Gephi (Version 0.10.1)<br>Statistical analysis: SPSS Statistics (Version 26)<br>Details were described in Materials and Methods section.

For manuscripts utilizing custom algorithms or software that are central to the research but not yet described in published literature, software must be made available to editors and reviewers. We strongly encourage code deposition in a community repository (e.g. GitHub). See the Nature Portfolio guidelines for submitting code & software for further information.

Data

Policy information about availability of data

All manuscripts must include a data availability statement. This statement should provide the following information, where applicable:

- Accession codes, unique identifiers, or web links for publicly available datasets
- A description of any restrictions on data availability
- For clinical datasets or third party data, please ensure that the statement adheres to our policy

The sequencing data has been deposited in the NCBI database named BioProject PRJNA1059279.

Research involving human participants, their data, or biological material

Policy information about studies with human participants or human data. See also policy information about sex, gender (identity/presentation), and sexual orientation and race, ethnicity and racism.

Reporting on sex and gender	No human research participants involved in this study.
Reporting on race, ethnicity, or other socially relevant groupings	No human research participants involved in this study.
Population characteristics	No human research participants involved in this study.
Recruitment	No human research participants involved in this study.
Ethics oversight	No human research participants involved in this study.

Note that full information on the approval of the study protocol must also be provided in the manuscript.

Field-specific reporting

Please select the one below that is the best fit for your research. If you are not sure, read the appropriate sections before making your selection.

☐ Life sciences ☐ Behavioural & social sciences ☒ Ecological, evolutionary & environmental sciences

For a reference copy of the document with all sections, see nature.com/documents/nr-reporting-summary-flat.pdf

Ecological, evolutionary & environmental sciences study design

All studies must disclose on these points even when the disclosure is negative.

Study description	This study investigated the fouling behavior of RO membranes in the treatment of desulfurization wastewater. The wastewater was treated with a coagulation pretreatment using $FeCl_3$ (residual Fe = 2.59 mg/L) and $AlCl_3$ (residual Al = 2.36 mg/L), as well as a control group without coagulant. Following coagulation, the supernatant was filtered through a 0.45 μm microfiltration membrane before being fed into an RO system. The RO filtration tests were conducted under constant pressure (~2.0 MPa) and cross-flow velocity (~6 cm/s), with samples collected at multiple intervals over a 20-day period.
Research sample	The samples were desulfurization wastewater and RO membranes used to study fouling behavior under industrial conditions.
Sampling strategy	Desulfurization wastewater was treated with a coagulation unit, microfiltration system, and RO system. $FeCl_3$ and $AlCl_3$ were used as coagulants, along with a control group. Membrane and foulant samples were collected after each treatment. During RO filtration, membrane flux was measured periodically under constant conditions (2.2 MPa), with samples taken at intervals (i.e., 0.25, 0.5, 1, 2, 4, 6, 8, 10, 12, 15, and 20 d) to monitor fouling development.
Data collection	The experiment data were mainly collected by the first author, Haojie Ding, under the supervision of Prof. Xia Huang. The fouling behavior, membrane flux, and water quality parameters were were continuously monitored throughout the experimental period.
Timing and spatial scale	The experiment data collection was conducted in the laboratory from February 2023 to July 2023.
Data exclusions	No data were excluded from the analyses.
Reproducibility	All attempt to repeat the experiment were successful. The results can be reproduced with the same materials and experimental conditions.
Randomization	Not applicable to our study.

Blinding	Not applicable to our study.

Did the study involve field work? ☐ Yes ☒ No

Reporting for specific materials, systems and methods

We require information from authors about some types of materials, experimental systems and methods used in many studies. Here, indicate whether each material, system or method listed is relevant to your study. If you are not sure if a list item applies to your research, read the appropriate section before selecting a response.

Materials & experimental systems

n/a	Involved in the study
☒	☐ Antibodies
☒	☐ Eukaryotic cell lines
☒	☐ Palaeontology and archaeology
☒	☐ Animals and other organisms
☒	☐ Clinical data
☒	☐ Dual use research of concern
☒	☐ Plants

Methods

n/a	Involved in the study
☒	☐ ChIP-seq
☒	☐ Flow cytometry
☒	☐ MRI-based neuroimaging

Plants

Seed stocks	No seed stocks or other plant materials were involved in this study.
Novel plant genotypes	No novel plant genotypes were involved in this study.
Authentication	No authentication procedures for any seed stocks or novel genotypes were involved in this study.
